# Exogenous Melatonin Attenuates Sleep Restriction-Induced Kidney Injury via Gut Microbiota-Derived Propionate in Mice

**DOI:** 10.3390/antiox14101218

**Published:** 2025-10-09

**Authors:** An Cui, Qingyun Guan, Zixu Wang, Jing Cao, Yulan Dong, Yaoxing Chen

**Affiliations:** National Key Laboratory of Veterinary Public Health and Safety, College of Veterinary Medicine, China Agricultural University, Haidian, Beijing 100193, China; cuian20211224@163.com (A.C.);

**Keywords:** melatonin, sleep restriction, kidney injury, gut microbiota, propionic acid

## Abstract

Chronic sleep restriction (SR) impairs multiple organs. Although exogenous melatonin counteracts SR-induced gut microbiota disruption, its role in protecting renal function and the involvement of gut microbiota remain unclear. To this end, we subjected mice to a 28-day SR paradigm with exogenous melatonin treatment or antibiotic-induced microbiota depletion. SR mice demonstrated significant renal dysfunction evidenced by elevated serum creatinine, blood urea nitrogen, and uric acid levels compared to controls. Histopathological analysis revealed characteristic tubular abnormalities in SR mice, including epithelial degeneration and lumen dilation, with reduced expression of key renal filtration markers (*Nephrin*, *Podocin*, *CD2-associated protein*, and *α-Actinin-4*). All of these could be mitigated by melatonin treatment, and all changes were statistically significant (*p* < 0.05 or *p* < 0.01). Intriguingly, microbiota depletion significantly reversed the protective effect of exogenous melatonin on kidney injury in SR mice, while propionic acid supplementation mitigated SR-induced kidney injury. Furthermore, we found that gut microbiota and the metabolite propionic acid mediated the role of exogenous melatonin probably through attenuating SR-induced renal oxidative damage, including regulating renal superoxide dismutase (SOD) activity, total antioxidant capacity (T-AOC), and malondialdehyde (MDA) level. These findings collectively indicated that melatonin may ameliorate SR-associated kidney injury through gut microbiota-derived propionic acid. Our finding highlights a novel gut–kidney axis in SR-related pathophysiology.

## 1. Introduction

Sleep is essential for maintaining systemic homeostasis across species. In contemporary society, chronic sleep restriction (≤6 h/day) affects over 30% of adults globally, primarily driven by escalating occupational demands and digitalization-induced lifestyle changes [1]. This behavioral adaptation induces multiorgan dysfunction through circadian disruption and oxidative stress pathways [2]. While acute sleep deprivation impairs cognitive performance and emotional regulation [3], chronic restriction progressively damages vital organs, notably contributing to hepatic steatosis, cardiovascular remodeling [4]. Emerging evidence further implicates SR in renal pathophysiology, though the underlying molecular mechanisms remain enigmatic [2]. As organs with high energy demands, kidneys exhibit heightened susceptibility to chronic oxidative injury [5]. Renal impairment manifests through progressive stages from acute kidney injury (AKI) to chronic kidney disease (CKD), with interstitial fibrosis representing a terminal pathological hallmark [6]. Research has shown that short sleep duration was associated with higher uric acid (UA) levels [7]. A study using an aged rat sleep deprivation model revealed that three days of sleep restriction caused hepatic steatosis, myocardial fiber dissolution, and renal cast formation [8]. After five days, hepatic lipoidosis, renal tubular epithelial cell apoptosis, and myocardial fibrosis emerged [8]. This indicates that sleep restriction exerts physiological and morphological effects on the kidneys. And sleep restriction during pregnancy is able to modify renal development, resulting in morphologic and functional alterations in young offspring [9]. This indicates that sleep restriction has a certain impact on the kidneys, but the specific details remain unclear. These diverse approaches highlight the necessity of studying disease-specific mechanisms, especially in the area of sleep-related kidney diseases, which are clinically relevant but understudied.

Melatonin (N-acetyl-5-methoxytryptamine) is a classic and well-recognized circadian rhythm-regulating molecule. Exogenous melatonin not only modulates sleep and mitigates the harm caused by sleep restriction but also exerts multifaceted renal protective effects through antioxidant, anti-inflammatory, and mitochondrial homeostasis regulation [10]. Previous studies validate its therapeutic potential against cadmium-induced tubular necrosis [11] and sepsis-associated glomerular injury [10]. Intriguingly, recent evidence positions melatonin as a microbiota modulator, capable of restoring gut barrier integrity [12] and microbial diversity, furthermore, following SR-induced gut microbiota dysbiosis, propionate metabolism is also affected [13] under circadian disruption. This unique convergence of oxidative stress mitigation and microbiota remodeling posits melatonin as a key modulator of gut–kidney crosstalk. Nevertheless, the therapeutic implications of this axis remain uncharted territory in sleep deprivation-associated nephropathy, particularly regarding fibroblast activation and tubular injury progression.

Emerging insights into microbial metabolites further strengthen this conceptual framework. Short-chain fatty acids, particularly propionate, demonstrate renal protective effects via G-protein-coupled receptor (GPR) signaling and histone deacetylase inhibition [14]. Notably, melatonin administration increases fecal propionate levels by 40% in sleep-disturbed models [13], implying a microbiota-dependent pathway. However, the triangular relationship between sleep restriction, melatonin, and microbial metabolites remains uncharacterized in renal pathophysiology.

Therefore, we established a 28-day mouse model of SR, and mice were treated with exogenous melatonin to investigate the protective role of melatonin in kidney injury affected by SR. Furthermore, the role of gut microbiota and its derived metabolites in melatonin intervention and SR-induced kidney injury was explored.

## 2. Materials and Methods

### 2.1. Animal Models

**Figure 1:**
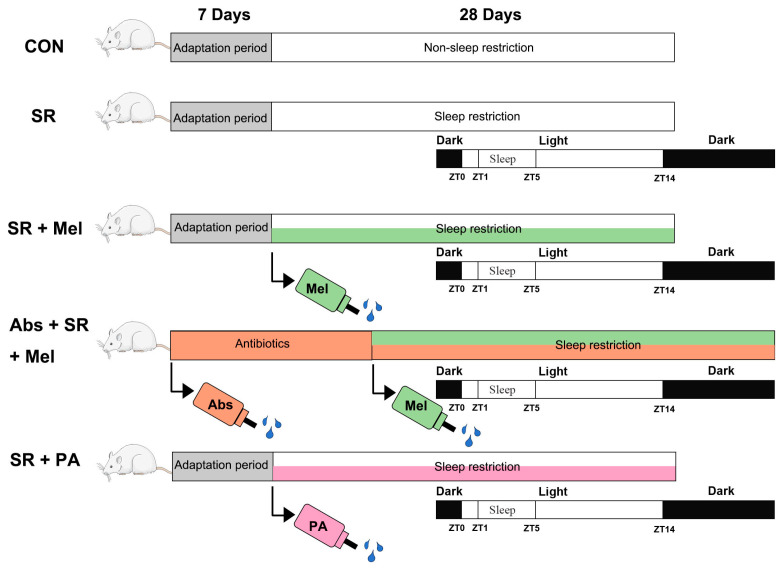
Animal testing procedures.

All procedures complied with the Care and Use of Laboratory Animals published by the Animal Welfare Committee of the Agricultural Research Organization, China Agricultural University (Approval No. AW18079102-2). All efforts were made to minimize animal suffering and to reduce the number of animals used, following the 3Rs principles (Replacement, Reduction, and Refinement). Ninety male ICR mice (8 weeks old, obtained from Vital River Laboratory Animal Technology Co., Ltd., Beijing, China) were acclimatized in a controlled environment with a 14:10 light-dark cycle for one week. Set the temperature in the rearing room to 22–25 °C, with a relative humidity of 50 ± 10%. All mice were randomly assigned to five experimental groups using a computer-generated random number table to ensure unbiased allocation: a SR (*n* = 24), a SR plus melatonin supplementation group (SR + Mel, *n* = 24), an intestinal microbiota depletion plus SR and melatonin supplementation group (Abs + SR + Mel, *n* = 8), a SR plus propionic acid supplementation group (SR + PA, *n* = 8), and a non-SR control group (CON, *n* = 24). The minimal sample size was determined using a power analysis with a power of 0.8 and an α of 0.05, resulting in n = 8 per group. During the experiment, 3 mice were excluded from the final analysis due to death during the experiment (*n* = 3). Sleep restriction was imposed on the designated groups for four weeks using the modified multiple platform method as previously described [13]. Set up the test procedure as shown in Figure 1. Zeitgeber time (ZT) zero was set at 7:00 a.m. SR spans from ZT5 of the current day to ZT1 of the following day, while sleep occurs from ZT1 to ZT5 of the same day. This means that mice are subjected to 20 h of sleep restriction daily, with only 4 h allocated for actual sleep. As described in previous studies [13], a modified multi-platform method was used as the sleep deprivation device. This device consists of a water tank (320 mm × 260 mm × 170 mm) and 12 cylindrical platforms (diameter 30 mm) spaced 4 cm apart. Feeders and waterers are placed inside, allowing mice to eat and drink normally. Six mice were placed in the MMPM device, and tap water was added until the water level was 4 cm below the cylindrical platforms. Mice could move between platforms and eat, but when they entered the rapid eye movement (REM) stage of sleep, relaxed muscles could cause them to fall into the water, thereby awakening them.

In the SR + Mel group, mice were administered melatonin (M5250; Sigma, St. Louis, MO, USA) via drinking water at a concentration of 10^−5^ mol/L. Melatonin was dissolved in absolute ethanol to achieve a final concentration of 0.12% absolute ethanol. The control group, which did not receive melatonin supplementation, was given an equivalent volume of 0.12% absolute ethanol in drinking water.

Mice were administered oral antibiotics to deplete their microbiota and were subsequently supplemented with propionic acid via their drinking water. The antibiotics utilized were 1.0 g/L ampicillin and 0.5 g/L neomycin [15]. The concentration of the supplemented propionic acid was 0.6% [16]. The specific dosing schedule and experimental design are shown in Figure 1. On the morning of day 29 at ZT1, five mice from each group were anesthetized with 10% chloral hydrate and subsequently euthanized. Plasma and kidney samples were collected for histological and biochemical analyses.

### 2.2. Blood Biochemical Analysis

We utilized reagent kits supplied by the Nanjing Jiancheng Bioengineering Institute (Nanjing, China) to evaluate several blood biochemical parameters. The specific indicators assessed were: glucose (GLU, A1541-1-1), total cholesterol (T-CHO, A111-1-1), aspartate aminotransferase (AST/GOT, C010-2-1), albumin (A028-2-1), total protein (TP, A045-4), triglyceride (TG, A110-1-1), creatinine (CRE, C011-2-1), blood urea nitrogen (BUN, C013-2-1), and uric acid (UA, C012-2-1). Each sample was analyzed in triplicate to ensure data reliability.

### 2.3. Histological Analysis

The harvested renal tissues were fixed in 0.1 mol/L phosphate-buffered saline (pH 7.4, 4 °C) containing 4% paraformaldehyde for 48 h, followed by dehydration and paraffin embedding to prepare 5 μm thick sections. Hematoxylin–eosin (H&E; G1120, Solarbio Life Sciences, Beijing, China) and periodic acid–Schiff (PAS; G1281, Solarbio Life Sciences, Beijing, China) staining were performed to evaluate the extent of kidney injury. Additionally, Masson trichrome and Sirius red staining were conducted to assess renal fibrosis. Microscopic images were captured at 400× magnification with a scale bar of 50 µm from at least 30 randomly selected areas across four independent renal samples using an Olympus BX51 microscope (Olympus, Tokyo, Japan). Data analysis was conducted using Image J 1.54h software (Media Cybernetics, Rockville, MD, USA).

### 2.4. RNA Isolation and Quantification Real-Time (qRT)-PCR

Total RNA was extracted from kidney tissues (*n* = 5) using Trizol reagent (CW0580, CoWin Biotech Co., Ltd., Beijing, China) according to the manufacturer’s protocol. A total of 1000 ng of RNA was reverse transcribed into a 20 µL cDNA reaction volume using the HiScript II First Strand cDNA Synthesis Kit (R212-01, Vazyme Biotech Co., Ltd., Nanjing, China). The reverse transcription conditions were set at 50 °C for 15 min and 85 °C for 2 min Quantitative real-time PCR amplification of target genes and internal reference genes was performed using AceQ qPCR SYBR Green Master Mix (Q111-02, Vazyme Biotech Co., Ltd., Nanjing, China) on the StepOne real-time PCR system (Applied Biosystems, Boston, MA, USA), with three technical replicates. Amplification parameters included: 95 °C for 5 min (initial denaturation); 40 cycles of 95 °C for 10 s (denaturation) and 60 °C for 30 s (annealing/extension). Relative mRNA expression levels were determined using the 2^−∆∆Ct^ method and normalized to the expression level of Gapdh. Primer sequences are provided in Table A1.

### 2.5. Determination of Antioxidant Activity and Lipid Peroxidation

We employed commercially available kits (Beyotime Biotechnology Co., Ltd., Shanghai, China) to assess the antioxidant capacity through the measurement of total superoxide dismutase (SOD) activity (S0101S), total antioxidant capacity (T-AOC) (S0116), and lipid peroxidation levels via malondialdehyde (MDA) content (S0131S). Each sample was analyzed in triplicate.

### 2.6. Statistical Analysis

Mice were randomly allocated to each experimental group to ensure the balance across all mouse studies. Data are expressed as mean ± standard error of the mean (SEM). Normality was assessed using the Shapiro–Wilk test, and homogeneity of variances was confirmed with the Brown–Forsythe test. Statistical significance was assessed using two-way analysis of variance (ANOVA) for comparisons among three or more groups. *p* < 0.05 was regarded as indicating a significant difference. All statistical analyses were conducted using GraphPad Prism software, version 10.0 (GraphPad Software, La Jolla, CA, USA).

## 3. Results

### 3.1. Chronic SR Caused Kidney Injury and Fibrosis in Mice

**Figure 2:**
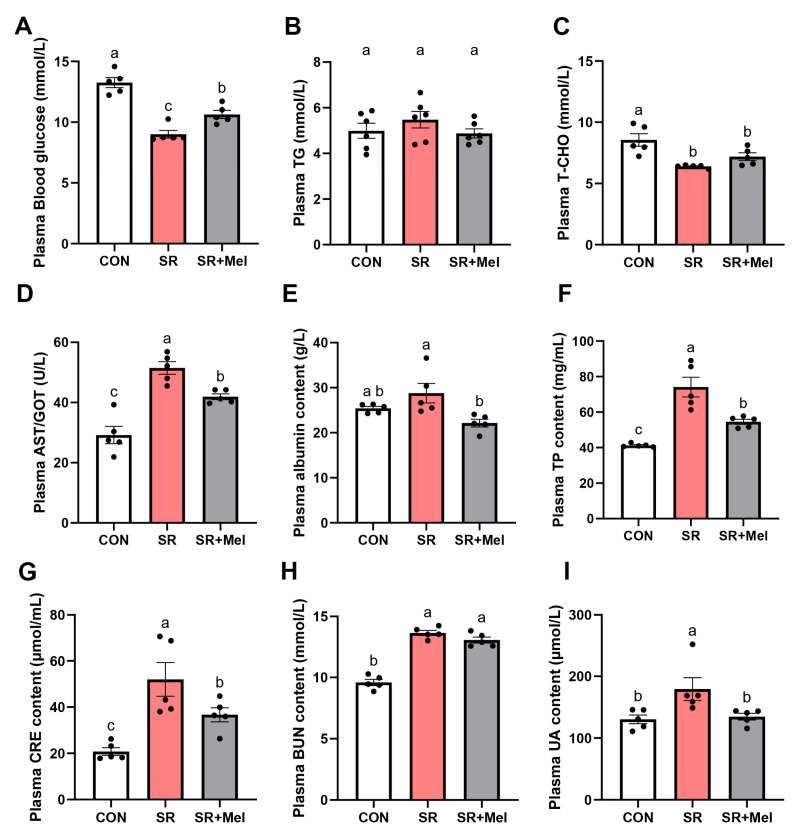
Exogenous melatonin ameliorates sleep restriction-induced renal impairment in mice. Plasma levels of (**A**) GLU changes; (**B**) TG; (**C**) T-CHO; (**D**) AST/GOT; (**E**) albumin; (**F**) TP; (**G**) CRE; (**H**) BUN; (**I**) UA in mice (*n* = 5). CON: non-sleep-restricted control group; SR: sleep-restricted group; SR + Mel: sleep-restricted group with melatonin supplementation. Data represent mean ± SEM (*n* = 5 mice per group). Groups labeled with different letters signify statistically significant differences (*p* < 0.05).

To evaluate the impact of SR on renal pathophysiology, we systematically analyzed biochemical, histological, and molecular markers in mice. Initial plasma profiling revealed significantly reduced GLU (Figure 2A) and T-CHO (Figure 2C) levels in the SR group, alongside a non-significant trend of elevated TG (Figure 2B). A marked increase in AST/GOT levels (Figure 2I) suggested SR-induced metabolic perturbations. Concurrently, SR mice exhibited significant elevations in indicators of renal functional impairment including TP, albumin, CRE, BUN, and UA (Figure 2D–H).

**Figure 3:**
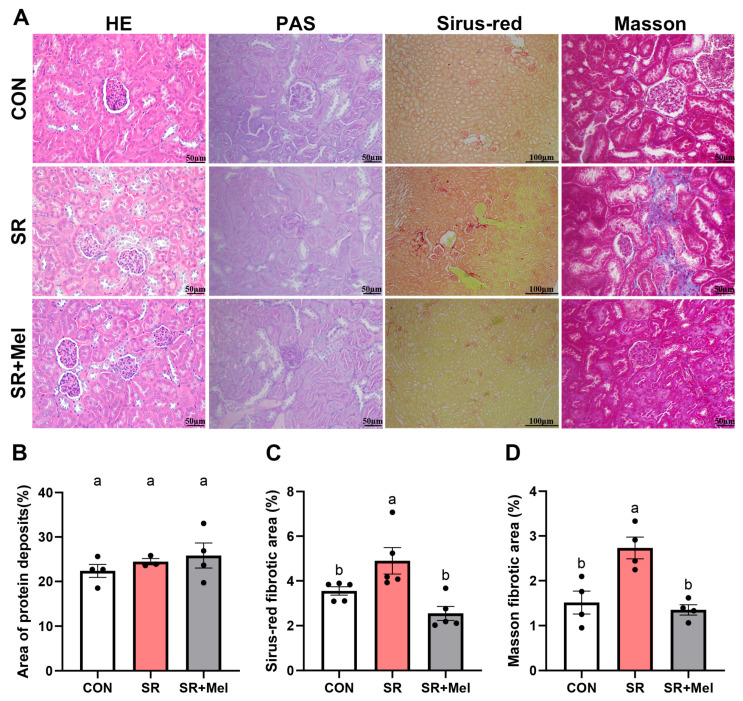
Exogenous melatonin attenuates sleep restriction-induced renal fibrosis in mice. (**A**) HE staining (scale bar: 50 μm), PAS staining (scale bar: 50 μm), Sirius red staining (scale bar 100 μm) and Masson staining (scale bar 50 μm) in kidneys; (**B**) Quantification of PAS-positive areas; (**C**) Positive area of Sirius red staining; (**D**) Positive areas of Masson staining. CON: non-sleep-restricted control group; SR: sleep-restricted group; SR + Mel: sleep-restricted group with melatonin supplementation. Data represent mean ± SEM (*n* = 4 mice per group). Groups labeled with different letters signify statistically significant differences (*p* < 0.05).

Morphological analysis via HE and PAS staining demonstrated intact glomerular and tubular structures in controls, characterized by clear lumens and uniform epithelial cell staining (Figure 3A). In contrast, SR mice displayed narrowed glomerular spaces, proximal tubule dilation, and minor protein deposition, though these changes lacked statistical significance (Figure 3B). The statistical results of Sirius red staining (Figure 3A,C) and Masson staining (Figure 3A,D) further consistently revealed pronounced renal interstitial collagen deposition, indicating apparent fibrosis.

**Figure 4:**
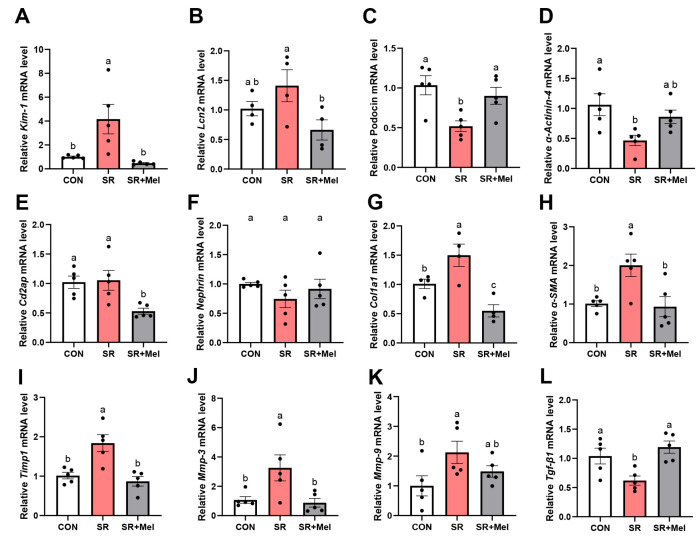
Exogenous melatonin attenuates sleep restriction-induced renal structural damage and fibrotic progression in mice. Quantitative relative mRNA levels of (**A**) *Kim-1*; (**B**) *Lcn2*; (**C**) *Podocin*; (**D**) *α-Actinin-4*; (**E**) *Cd2ap*; (**F**) *Nephrin*; (**G**) *Col1α1*; (**H**) *α-SMA;* (**I**) *Timp1*; (**J**) *Mmp-3*; (**K**) *Mmp-9*; (**L**) *Tgf-β1*. CON: non-sleep restriction control group; SR: sleep restriction group; SR + Mel: Sleep restriction + melatonin supplementation group. Data represent mean ± SEM (*n* = 5 mice per group). Groups labeled with different letters signify statistically significant differences (*p* < 0.05).

qRT-PCR analysis further showed that SR mice exhibited upregulated expression of kidney injury biomarkers (*Kim-1*) (Figure 4A) and fibrosis-associated genes (*Col1α1*, *α-SMA*) (Figure 4G,H), alongside diminished mRNA levels of podocyte structural proteins (*Podocin*, *α-actinin-4*) (Figure 4C,D). Elevated expression of matrix metalloproteinases (*Mmp3*, *Mmp9*) (Figure 4J,K) and tissue inhibitors (*Timp1*) (Figure 4I) suggested dysregulated extracellular matrix (ECM) turnover. Paradoxically, *Tgf-β1*, a pro-fibrotic cytokine, was significantly downregulated in SR mice (Figure 4L). No significant difference was observed in *Lcn2* (Figure 4B), *Cd2ap* (Figure 4E) or *Nephrin* expression (Figure 4F).

Collectively, these data demonstrate that SR induces renal functional impairment, structural damage, and fibrotic progression via dysregulation of injury biomarkers, ECM remodeling factors, and podocyte integrity markers.

### 3.2. Exogenous Melatonin Improves SR-Induced Kidney Injury and Fibrosis in Mice

To delineate the therapeutic role of exogenous melatonin, we compared the SR + Mel group with SR or control cohorts. Plasma biochemical analysis demonstrated that melatonin significantly reversed SR-induced hypoglycemia (Figure 2A) and normalized elevated albumin and AST/GOT levels (Figure 2E,I), though it exhibited no significant effects on T-CHO or TG (Figure 2B,C). Melatonin markedly attenuated SR-elevated CRE and UA levels but showed limited efficacy against BUN (Figure 2F–H), indicating partial restoration of renal function.

Histopathological evaluation via HE, PAS, Sirius red, and Masson staining revealed that melatonin ameliorated SR-induced structural damage, reducing glomerular narrowing, tubular dilation, and interstitial collagen deposition (Figure 3A–D).

At the molecular level, it showed that melatonin significantly downregulated kidney injury biomarkers (*Kim-1*, *Lcn2*) (Figure 4A,B) and fibrosis-associated genes (*Col1α1*, *α-SMA*) (Figure 4G,H), while partially restoring *Cd2ap* expression (Figure 4E). Concurrently, melatonin suppressed extracellular matrix regulators, reducing *Timp1* (Figure 4I) and *Mmp3* (Figure 4J) levels. Paradoxically, melatonin upregulated *Tgf-β1* (Figure 4L). Meanwhile, melatonin failed to rescue mRNA levels of podocyte integrity markers (*Podocin*, *α-actinin-4*, *Nephrin*) (Figure 4C,D,F) and *Mmp9* (Figure 4K).

Collectively, exogenous melatonin partially mitigates SR-induced renal dysfunction by modulating injury biomarkers, ECM remodeling, and fibrotic pathways, though its inability to restore podocyte structural proteins.

### 3.3. Gut Microbiota-Derived Propionic Acid Mediated the Protective Role of Exogenous Melatonin in Kidney Injury Induced by SR in Mice

**Figure 5:**
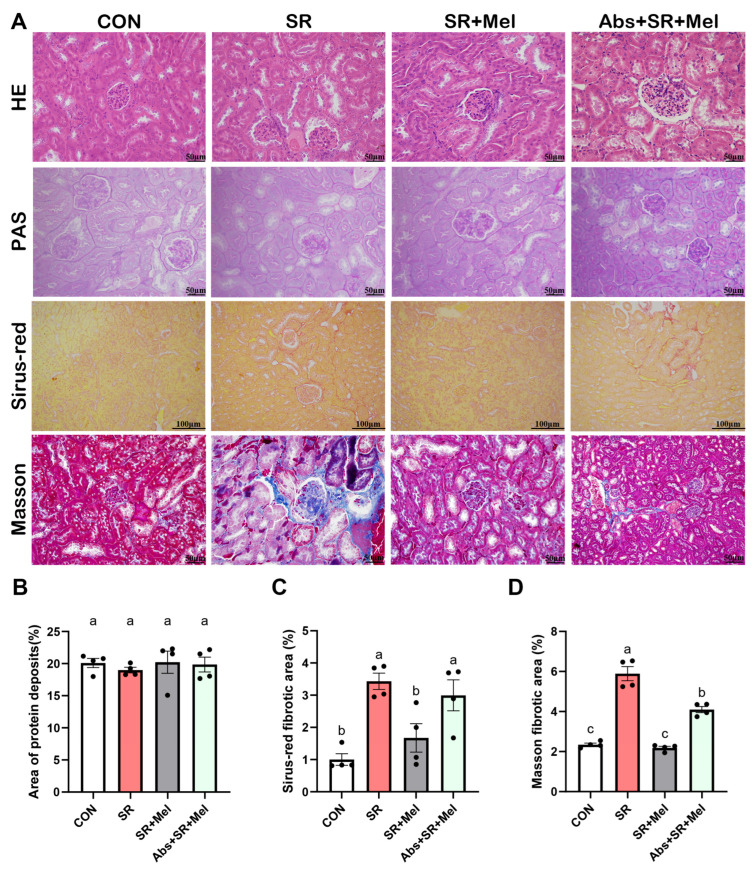
The impact of intestinal microbiota depletion on exogenous melatonin’s mitigation of sleep restriction-induced kidney injury and fibrosis in mice. (**A**) HE staining, PAS staining (scale bar: 50 μm), Sirius red staining (scale bar 100 μm) and Masson staining (scale bar 50 μm) in kidneys; (**B**) Positive area of PAS staining; (**C**) Positive area of Sirius red staining; (**D**) Positive areas of Masson staining. CON: non-sleep-restricted Control group; SR: sleep-restricted group; SR + Mel: sleep-restricted with melatonin supplementation group; Abs + SR + Mel: intestinal microbiota depleted, sleep-restricted with melatonin supplementation group. Data represent mean ± SEM (*n* = 4 mice per group). Groups labeled with different letters signify statistically significant differences (*p* < 0.05).

The elimination of gut microbiota has been proven to alleviate colonic and renal damage [17]. We hypothesized that the gut microbiota might mediate the protective effect of exogenous melatonin on kidney injury induced by SR in mice. HE staining showed that melatonin treatment significantly alleviated the disappearance of the renal capsule structure, dilation of renal tubules, and detachment of renal tubular epithelial cells forming casts induced by SR; however, the absence of gut microbiota reversed the improvement effect of melatonin (Figure 5A). PAS staining was employed to evaluate protein deposition, revealing no significant differences among the groups (Figure 5A,B). Sirius red staining (Figure 5A,D) and Masson staining (Figure 5A,C) results consistently indicated that melatonin treatment significantly ameliorated renal fibrosis in the SR group, whereas antibiotic-induced microbiota depletion abolished its protective effects.

**Figure 6:**
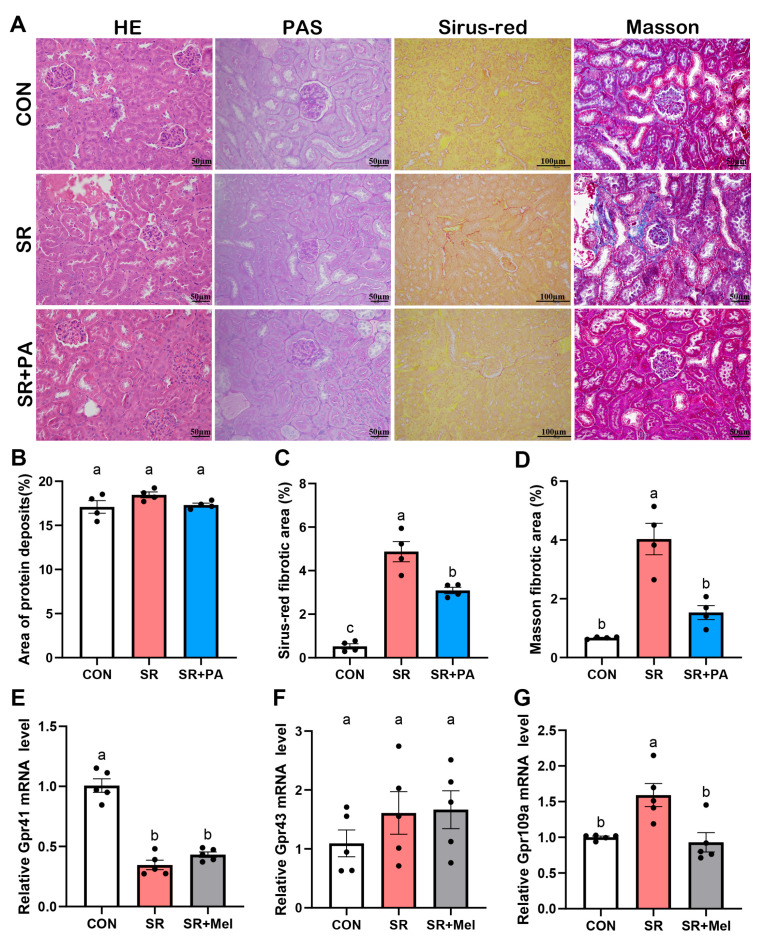
The administration of propionic acid significantly mitigates kidney injury and the fibrosis process in mice induced by sleep restriction. (**A**) HE staining, PAS staining (scale bar: 50 μm), Sirius red staining (scale bar 100 μm) and Masson staining (scale bar 50 μm) in kidneys; (**B**) Positive area of PAS staining; (**C**) Positive area of Sirius red staining; (**D**) Positive areas of Masson staining. Quantitative relative mRNA levels of (**E**) *Gpr41*; (**F**) *Gpr43*; (**G**) *Gpr109a* in the kidney. CON: non-sleep restriction control group; SR: sleep restriction group; SR + Mel: sleep restriction with melatonin supplementation group; SR + PA: sleep restriction with propionic acid supplementation group. Data represent mean ± SEM (*n* = 4 mice per group). Groups labeled with different letters signify statistically significant differences (*p* < 0.05).

The gut microbiota predominantly mediates its physiological effects via microbial metabolites. Our previous work demonstrated that exogenous melatonin significantly restored the gut microbes that produce propionic acid affected by SR [13]. Since propionic acid could effectively mitigate kidney injury [14], we incorporated a propionate supplementation group to investigate its role in SR-induced kidney injury. Compared with the SR group, propionic acid treatment significantly mitigated the shedding of renal tubular epithelial cells but did not significantly affect protein deposition (Figure 6A,B). Besides, propionic acid effectively alleviated renal interstitial fibrosis induced by SR and reduced collagen accumulation in the renal interstitium (Figure 6A,C,D). Further, we assessed the mRNA expression levels of propionic acid receptors in kidney tissue. The findings indicated a decrease in *Gpr41* expression in the SR group, while melatonin had no significant impact on its expression (Figure 6E). No significant differences were observed in *Gpr43* expression among the groups (Figure 6F). Notably, *Gpr109a* expression was elevated in the SR group but exhibited a decreasing trend in the melatonin-treated group (Figure 6G).

### 3.4. Gut Microbiota-Derived Propionic Acid Mediated the Protective Effect of Melatonin on SR by Inhibiting Renal Oxidative Damage

**Figure 7:**
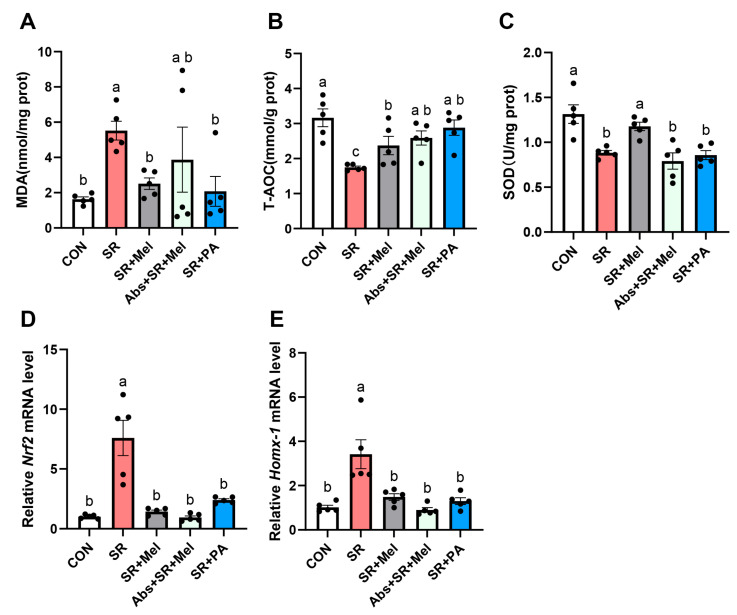
Gut microbiota-derived propionic acid mediated the protective effect of melatonin on SR by inhibiting renal oxidative damage Kidney (**A**) MDA; (**B**) SOD and (**C**) T-AOC levels. Quantitative relative mRNA levels of (**D**) *Nrf2* and (**E**) *Hmox-1* in the kidney. CON: non-sleep restriction control group; SR: sleep restriction group; SR + Mel: Sleep restriction + melatonin supplementation group; Abs + SR + Mel: intestinal microbiota depleted, sleep-restricted with melatonin supplementation group; SR + PA: sleep restriction with propionic acid supplementation group. Data represent mean ± SEM (*n* = 5 mice per group). Groups labeled with different letters signify statistically significant differences (*p* < 0.05).

Given the established role of oxidative stress in kidney injury, it remains to be determined whether gut microbiota-derived propionic acid mediates the protective effects of melatonin against sleep restriction by attenuating renal oxidative stress. To this end, we quantified oxidative stress markers in kidney, including T-AOC activity, SOD activity, and MDA levels. Compared with CON, SR mice exhibited elevated MDA levels (Figure 7A), a product of oxidative stress, and significantly reduced T-AOC (Figure 7B) and diminished SOD activity (Figure 7C). Notably, melatonin treatment restored T-AOC activity, enhanced SOD activity, and normalized MDA levels to baseline (Figure 7A–C), demonstrating its potent antioxidative properties in mitigating SR-induced oxidative stress. However, antibiotic treatment reversed the protective role of melatonin, including reduced T-AOC, impaired SOD activity, and elevated MDA (Figure 7A–C). In addition, propionic acid supplementation significantly reversed SR-induced oxidative damage (Figure 7A–C).

To assess oxidative stress modulation in renal tissues, we quantified mRNA levels of nuclear factor erythroid 2-related factor 2 (*Nrf2*, Figure 7D) and heme oxygenase 1 (*Hmox-1*, Figure 7E). Compared with CON, SR significantly upregulated *Nrf2* and *Hmox-1* expression compared to control. Melatonin treatment partially reversed these SR-induced elevations, whereas antibiotic depletion or propionic acid interventions showed negligible effects. These findings demonstrate that exogenous melatonin may attenuate SR-mediated renal oxidative damage through gut microbiota-derived propionic acid.

## 4. Discussion

In this study, we found that chronic SR induces renal dysfunction and structural damage, particularly fibrosis; however, melatonin supplementation could partially restore renal morphology and function and mitigate fibrosis. The lack of change in PAS staining suggests that propionate’s beneficial effects are more closely linked to mitigating interstitial fibrosis and tubular injury than to reducing proteinaceous casts. Mechanistically, gut microbiota and the metabolite propionic acid may mediate the protective role of melatonin in SR-induced kidney injury, which was probably through attenuating SR-induced renal oxidative damage.

Previous studies have demonstrated that SR can lead to renal dysfunction [2]. Previous laboratory studies have demonstrated that supplemental exogenous melatonin administered via drinking water can restore plasma melatonin levels in mice, thereby validating the efficacy of our research findings [18]. We observed that SR induced varying degrees of damage to the overall health of mice, as evidenced by serum metabolite profiles, with renal impairment being particularly pronounced. Specifically, SR led to a significant increase in serum CRE, BUN, and UA levels. The serum concentrations of CRE, BUN, and UA serve as biomarkers for assessing the extent of renal tubular injury [19]. Elevated levels of these markers suggest compromised renal tubular function. Furthermore, our analyses at both the morphological and transcriptional levels substantiated this observation. The expression of *Kim-1*, a well-established marker of kidney injury [20], was markedly elevated in the SR group. Melatonin’s renal protective effects appear to be primarily mediated through the attenuation of inflammatory and fibrotic pathways, as evidenced by the reduction of *Kim-1*, *Lcn2*, and collagen genes. Molecular markers associated with glomeruli and renal tubules, including *Nephrin*, *Podocin*, *Cd2ap*, and *α-Actinin-4*, were also down-regulated in the kidney of SR mice. The selective reduction of *Podocin* and *α-actinin-4*, but not *Nephrin* or *Cd2ap*, suggests a partial and specific injury to the podocyte cytoskeleton and slit diaphragm complex, rather than a global podocyte loss. This indicates variability in the susceptibility of different podocyte markers to SR-induced stress. Injured renal tubules can continuously amplify profibrotic signal transduction via multiple pathways, consequently resulting in myofibroblast transformation, proliferation, and fibrosis [21]. Unlike sepsis-induced AKI characterized by acute tubular necrosis [22], our model demonstrates chronic interstitial fibrosis, indicating distinct pathophysiological mechanisms in sleep-related nephropathy. The abnormal downregulation of *Tgf-β1* in SR may indicate the presence of a compensatory feedback mechanism attempting to counteract the ongoing fibrotic process, or our detection time may not have coincided with the peak expression of *Tgf-β1*, despite the fibrotic pathology still being present.

Melatonin is an endogenous hormone with extensive regulatory effects, which is crucial for sleep. In the present study, we found that exogenous melatonin could effectively attenuate SR-mediated kidney injury. Melatonin has demonstrated efficacy in reversing fibrosis across various renal fibrosis models. As reported, melatonin can prevent the *Tgf-β1*-induced transformation of renal interstitial fibroblasts into myofibroblasts by inhibiting reactive oxygen species-dependent mechanisms [23]. In chronic kidney disease, pretreatment with melatonin enhances the engraftment of bone marrow-derived mesenchymal stem cells into damaged renal tissue, thereby promoting renal regeneration [24]. Melatonin possesses the potential to inhibit cytoskeletal reorganization and restore mitochondrial function, through which it suppresses fibrosis [25]. The observed upregulation of *Tgf-β1* following melatonin treatment, despite an overall reduction in fibrosis, may seem counterintuitive. This could be explained by the dual role of *Tgf-β1* in both inflammation and repair, or by cell-type-specific effects where melatonin modulates *Tgf-β1* signaling in immune cells versus fibroblasts. Increasing evidence demonstrates that dysbiosis of the gut microbiota is prevalent among patients with CKD and diabetic kidney disease. The dysbiosis of the intestinal microbiota in CKD serves as a critical indicator of renal function impairment and disease progression [26]. Fecal microbiota transplantation thus emerges as a promising therapeutic alternative for restoring gut microbial balance and treating CKD [27]. We showed that antibiotic-induced microbiota depletion abolished the protective effects of melatonin treatment on SR-induced renal damage. The depletion of the gut microbiota markedly diminished the expression levels of markers associated with tubulointerstitial fibrosis [28]. Microbial metabolites may function as a critical intermediary between the gut microbiota and mitochondrial processes [29], and there was a significant interaction between the gut microbiome, its metabolite short-chain fatty acids, and kidney diseases [30]. The findings of this study demonstrate that supplementation with the gut microbiota metabolite propionic acid can significantly mitigate kidney injury induced by SR. While we observed changes in *Gpr41* and *Gpr109a* expression, the precise mechanisms by which they mediate protection from propionate in this model are still unclear. We guessed *GPR109a*, a receptor for propionic acid, as a potential key mediator through which melatonin may exert its protective effects via propionic acid signaling. However, it needs further investigation.

The disruption of gut microbiota homeostasis is closely related to oxidative stress in the body. Oxidative stress, which has been identified as a key trigger for fibrosis, has garnered considerable attention recently [31,32]. Our research has demonstrated that SR led to renal lipid peroxidation, with MDA significantly increasing and the activity of antioxidant enzymes being markedly inhibited in the SR group. Melatonin supplementation could significantly reverse the promoting effect of SR on renal oxidative stress. Upon binding to its downstream target *Hmox-1*, the transcription factor *Nrf2* can counteract pro-oxidative conditions [33]. The increase in *Nrf2* and *Hmox-1* transcripts in SR mice likely represents a compensatory antioxidant response to elevated oxidative stress. Research has found that certain functional nutrients, when administered at low doses, synergize with neurosteroids to activate cellular stress resistance mechanisms, thereby activating the *Nrf2* antioxidant pathway [34,35]. This may partially explain the role of SR in its upregulation. The subsequent partial normalization of these levels by melatonin is consistent with a reduction in the oxidative burden, rather than a direct suppression of these protective pathways. Melatonin is widely recognized for its ability to exert potent antioxidative effects. However, rather than functioning solely as a direct free-radical scavenger, growing evidence suggests its primary role may be as an inducer of the endogenous antioxidant defense system [36]. Our data support this inductive paradigm, showing that melatonin treatment restored the activity of key antioxidant enzymes following sleep deprivation, suggesting a mechanism that enhances host capacity to mitigate oxidative stress. Thus, it is not surprising that melatonin treatment recovers from the insufficient renal oxidative stress caused by SR. Interestingly, depletion of gut microbiota effectively reverses the ameliorative effect of exogenous melatonin on SR-induced renal oxidative stress, while propionic acid supplementation partially inhibits the pro-oxidative effect of SR on renal oxidative stress. As reported, circulating propionate concentration was associated with oxidative damage [37]. It suggests that the renal protective effect of melatonin is not only dependent on its own antioxidant capacity, but also closely related to the balance of the gut microbiota microenvironment [38]. Thus, the novel finding of the melatonin–propionate axis extends beyond its classical antioxidant properties, revealing a microbiota-dependent pathway for renal protection.

It is important to note a limitation of this study. We did not assess the glomerular filtration rate (GFR) via 24-h urine collection, which is one of the gold standards for quantifying renal function. Our decision was based on the long duration of our model. Furthermore, metabolic cage housing for urine collection introduces significant stress that could confound results related to the gut–kidney axis. Nevertheless, the consistent improvements observed across multiple complementary parameters—including CRE, BUN, UA, fibrosis markers, and oxidative stress indicators—provide compelling evidence for the kidney injury of SR. Additionally, we did not establish an exogenous melatonin supplementation group as a control. This is because previous studies have demonstrated that outcomes following exogenous melatonin supplementation alone are largely consistent with those of the control group [11]. Consequently, we did not include this group, which represents another limitation of our study.

## 5. Conclusions

Chronic SR could lead to kidney injury and interstitial fibrosis, which can be effectively mitigated by exogenous melatonin administration. Mechanistically, propionic acid derived from the gut microbiota may mediate the protective effects of melatonin, potentially by attenuating SR-induced oxidative damage in the kidneys.

## Data Availability

All data generated or analyzed during this study are included in this published article.

## Abbreviations

The following abbreviations are used in this manuscript:

Abs	Antibiotics
AKI	Acute kidney injury
AST/GOT	Aspartate aminotransferase
BUN	Blood urea nitrogen
CKD	Chronic kidney disease
CRE	Creatinine
GLU	Glucose
IL	Interleukin
MDA	Malondialdehyde
Mel	Melatonin
PA	Propionic acid
PAS	Periodic acid–Schiff
SCFA	Short-chain fatty acids
SOD	Superoxide dismutase
SR	Sleep restriction
T-AOC	Total antioxidant capacity
T-CHO	Total cholesterol
TG	Triglyceride
TNF-α	Tumor necrosis factor-α
TP	Total protein
UA	Uric acid
ZT	Zeitgeber time

## Appendix A

**Table A1:** Sequences of primers used for RT-PCR.

Gene	Primer Sequence (5′→3′)	Product	Accession No.
*Kim1*	F: ACATATCGTGGAATCACAACGAC	114	XM_011248784.3
	R: ACTGCTCTTCTGATAGGTGACA		
*Lcn2*	F: TGCAGGTGGTACGTTGTGG	418	NM_008491.1
	R: TGTTGTCGTCCTTGAGGC		
*Podocin*	F: GTGTCCAAAGCCATCCAGTT	232	XM_006496684.3
	R: GTCTTTGTGCCTCAGCTTCC		
*α-Actinin-4*	F: GCCATCCAGGACATCTCTGT	189	XM_006540257.4
	R: CCGCAGCTTGTCATACTCAA		
*Cd2ap*	F: AGGAATTCAGCCACATCCAC	200	NM_009847.4
	R: TTGAGGGAAACAGTCCCAAC		
*Nephrin*	F: CCCCTCTATGATGAAGTACAAATGGA	213	XM_011250647.3
	R: GTACGGATTTCCTCAGGTCTTCT		
*Col1α1*	F: CATGTTCAGCTTTGTGGACCT	94	NM_007742.4
	R: GCAGCTGACTTCAGGGATGT		
*α-SMA*	F: GTCCCAGACATCAGGGAGTAA	102	NM_007392.3
	R: TCGGATACTTCAGCGTCAGGA		
*Timp1*	F: GCAACTCGGACCTGGTCATAA	226	NM_011593.3
	R: CGGCCCGTGATGAGAAACT		
*Mmp-3*	F: ACATGGAGACTTTGTCCCTTTTG	192	NM_010809.3
	R: TTGGCTGAGTGGTAGAGTCCC		
*Mmp-9*	F: GGACCCGAAGCGGACATTG	139	NM_013599.5
	R: CGTCGTCGAAATGGGCATCT		
*Tgf-β1*	F: CTCCCGTGGCTTCTAGTGC	133	XM_036152883.1
	R: GCCTTAGTTTGGACAGGATCTG		
*iNOS*	F: GTTCTCAGCCCAACAATACAAGA	127	NM_010927.4
	R: GTGGACGGGTCGATGTCAC		
*Nrf2*	F: TCTTGGAGTAAGTCGAGAAGTGT	140	NM_006164.5
	R: GTTGAAACTGAGCGAAAAAGGC		
*Hmox-1*	F: AAGCCGAGAATGCTGAGTTCA	100	NC_000074.7
	R: GCCGTGTAGATATGGTACAAGGA		
*Gpr41*	F: CTTCTTTCTTGGCAATTACTGGC	158	NM_001033316.2
	R: CCGAAATGGTCAGGTTTAGCAA		
*Gpr43*	F: CTTGATCCTCACGGCCTACAT	137	XM_011250554.4
	R: CCAGGGTCAGATTAAGCAGGAG		
*Gpr109a*	F: CTGGAGGTTCGGAGGCATC	243	NM_030701.3
	R: TCGCCATTTTTGGTCATCATGT		
*Gapdh*	F: CCGAGAATGGGAAGCTTGTC	232	XM_036165840.1
	R: TTCTCGTGGTTCACACCCATC		

**Table A2:** Glossary.

Noun	Definition
acute kidney injury (AKI)	Increase in CRE by ≥50% within 7 days or increase in CRE by ≥0.3 mg/dL (26.5 μmol/L) within 2 days or oliguria for ≥6 h [6]
chronic kidney disease (CKD)	GFR < 60 mL/min/1.73 m^2^, and Elevated marker of kidney damage (albuminuria is most common) [6]
